# Targeting the GPR119/incretin axis: a promising new therapy for metabolic-associated fatty liver disease

**DOI:** 10.1186/s11658-021-00276-7

**Published:** 2021-07-07

**Authors:** Jianan Zhao, Yu Zhao, Yiyang Hu, Jinghua Peng

**Affiliations:** 1 Institute of Liver Diseases, Shuguang Hospital Affiliated To Shanghai, University of Traditional Chinese Medicine, 528, Zhangheng Road, Shanghai, China; 2 Institute of Clinical Pharmacology, Shuguang Hospital Affiliated To Shanghai, University of Traditional Chinese Medicine, 528, Zhangheng Road, Shanghai, China; 3 Key Laboratory of Liver and Kidney Diseases, Shanghai University of Traditional Chinese Medicine), Ministry of Education, 528 Zhangheng Road, Pudong District, Shanghai, 201203 China; 4 Shanghai Key Laboratory of Traditional Chinese Clinical Medicine, 528, Zhangheng Road, Shanghai, China

**Keywords:** GPCR, GPR119, Incretins, Metabolic (dysfunction)-associated fatty liver disease, Liver disease

## Abstract

In the past decade, G protein-coupled receptors have emerged as drug targets, and their physiological and pathological effects have been extensively studied. Among these receptors, GPR119 is expressed in multiple organs, including the liver. It can be activated by a variety of endogenous and exogenous ligands. After GPR119 is activated, the cell secretes a variety of incretins, including glucagon-like peptide-1 and glucagon-like peptide-2, which may attenuate the metabolic dysfunction associated with fatty liver disease, including improving glucose and lipid metabolism, inhibiting inflammation, reducing appetite, and regulating the intestinal microbial system. GPR119 has been a potential therapeutic target for diabetes mellitus type 2 for many years, but its role in metabolic dysfunction associated fatty liver disease deserves further attention. In this review, we discuss relevant research and current progress in the physiology and pharmacology of the GPR119/incretin axis and speculate on the potential therapeutic role of this axis in metabolic dysfunction associated with fatty liver disease, which provides guidance for transforming experimental research into clinical applications.

## Introduction

Metabolic (dysfunction)-associated fatty liver disease (MAFLD) has become a major health problem in developed countries. It has become the first pandemic liver disease in China, and its prevalence rate is ballooning. It is a genetic stress disorder related to the environment and to obesity, hypertension, hyperlipidemia, and type 2 diabetes [1]. The lifestyle of patients with MAFLD has a direct effect on disease development; for example, host microbial environment disorders and endocrine and metabolic environment disorders driven by poor diet and exercise habits are important factors in the development of MAFLD. Currently, MAFLD is highly heterogeneous; thus, categorizing all patients with a diverse and differential array of disease drivers as patients with non-alcoholic fatty liver disease (NAFLD) can negatively impact clinical decision making. Therefore, NAFLD has been renamed as MAFLD [2–7], and MAFLD is used instead of NAFLD in this article. The latest diagnostic criteria for MAFLD are based on histology (biopsy sample), imaging or blood biomarker evidence of fat accumulation in the liver (hepatic steatosis) with one of the following three criteria: overweight/obesity, diagnosis of type 2 diabetes mellitus (T2DM), or evidence of metabolic dysregulation [8–10]. Most drugs currently on the market are focused on weight and diet control, but they may produce side effects; for example, pioglitazone may cause weight gain. In addition, orlistat has no significant effect on liver fibrosis, and surgical procedures may be traumatic. Therefore, more effective and safer medications are needed [11, 12].

The G protein-coupled receptor (GPCR) superfamily has many extensively studied members [13, 14]. GPR119 is a member of the GPCR superfamily. GPR119 activation has ligand-dependent dual effects: pancreatic secretion of insulin in a glucose-dependent manner and intestinal secretion of incretins (glucagon-like peptide-1 [GLP-1] and glucose-dependent insulinotropic peptide [GIP]) [15]. In addition, many studies have shown that the activation of GPR119 causes an increase in intracellular cyclic AMP (cAMP) levels and the release of incretins, including GLP-1, GIP, and glucagon-like peptide-2 (GLP-2) [16]. GLP-1 is a peptide secreted by human small intestinal L cells. It has regulatory effects on the gastrointestinal tract, blood sugar regulation, and improvement of insulin resistance, such as reducing dietary intake, increasing satiety, increasing gastrointestinal motility and prolonging the time of gastric emptying [17]. Habib et al. demonstrated that GLP-1 and peptide YY (PYY) are colocalized in L cells, suggesting that PYY is involved in reducing dietary intake [18]. Studies have shown that the fatty acid amide-induced activation of GPR119 on intestinal L cells may promote more focused and specific GLP-1/PYY activity, including inhibiting gastric emptying, regulating satiety, and inhibiting intestinal peristalsis [19]. GLP-2, a sister protein of GLP-1, is synthesized in the brain stem and released by intestinal L cells. It has the functions of promoting nutrient absorption, protecting the intestinal barrier, reducing intestinal permeability, and exerting anti-inflammatory effects [20–23]. GLP-2 can also reduce dietary intake, although the effect is less pronounced than that of GLP-1. Importantly, Hsieh J et al. found that GLP-2 can increase fat absorption through the stimulated CD36 pathway and can promote the release of chylomicrons, lipoprotein particles that transport exogenous hypertriglyceridemia (TG), and ultimately promote lipolysis and inhibit an increase in body weight, which is undoubtedly beneficial for patients with MAFLD [24].

GLP-1 is expressed in the body for a short time because dipeptidyl peptidase 4 (DPPIV) quickly decomposes it. Therefore, increasing the level of glucagon-like peptide or inhibiting its decomposition is of potential clinical significance for treating MAFLD. A recent study by Shuyong Zhang and others found that Gordonoside F, a steroid glycoside isolated from the African cactiform *Hoodia gordonii*, directly targets GPR119 to induce weight loss [25]. Even with slight weight loss, insulin resistance, abnormal blood glucose and blood pressure respond and improve quickly [21, 26]. Because GPR119 has significant advantages in blood glucose regulation, it has been a drug target to treat type 2 diabetes mellitus with many excellent results [27, 28]. Here, we update and discuss the potential therapeutic effect of the GPR119/incretin axis in MAFLD to provide a basis for the transformation of innovative clinical results of MAFLD.

### Gene and tissue distribution of GPR119

*GRP119* has been described in various studies and has many aliases, such as SNORF25, GPCR2, 19AJ, OSGPR116, and glucose-dependent insulinotropic receptor [29–34]. Robert Fredriksson et al. first determined that GPR119 is an orphan receptor in the rhodopsin family [35]. Akatoshi Soga et al. confirmed, for the first time, in 2005 that GPR119 is activated by lysophosphatidylcholine (LPC), indicating that GPR119 is a de-orphanized GPCR [36]. GPR119 pertains to the biogenic amine and MECA (melanocortin, endothelial differentiation gene, cannabinoid, and adenosine) cluster of receptors [35, 37]. The human *GPR119* gene is located on chromosome X at Xp26.1; it contains only one coding exon and encodes a protein of 335 amino acid bases. Also, the *GPR119* gene is predicted to be widely present in other mammals, including rats, mice, rabbits, horses, and cattle. Among them, human GPR119 shares 82%, 37% and 73.7% amino acid identity with mouse, fugu and rat GPR119, respectively [35].

**Fig. 1:**
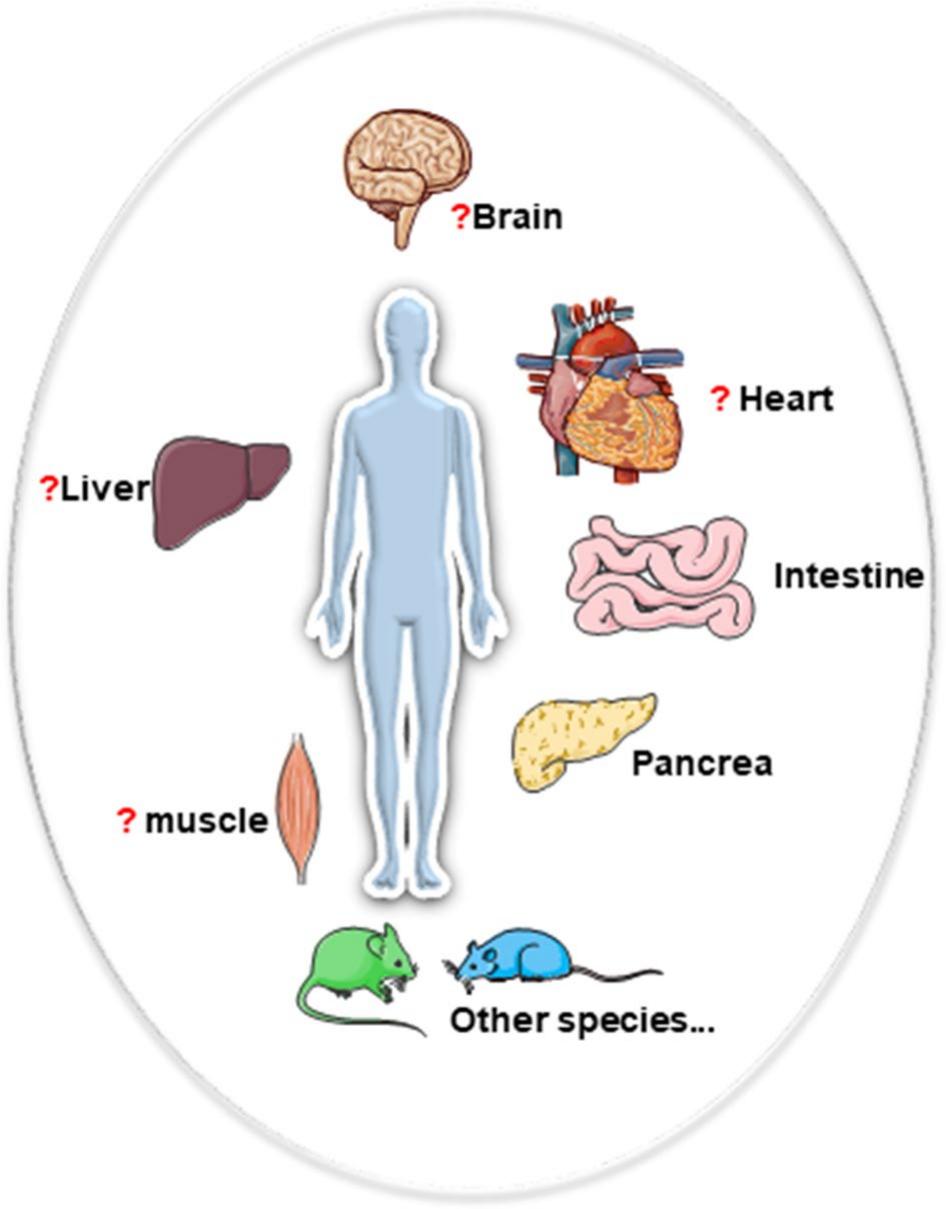
Tissue distribution of GPR119. GPR119 may be present in the brain, gastrointestinal digestive system, pancreas, liver, and heart, but there is still controversy (indicated with question marks in the figure). The reason may be low expression or differences in detection methods. In the future, some strict detections are still needed

In terms of tissue distribution, Zhi-l Ian GC and others found that compared with the entire pancreas, the expression of GRR119 is mainly distributed in the β cell fraction of the islet population, and it is also highly expressed in the gastrointestinal tract GLP-1-producing cells and GIP-producing cells, such as intestinal endocrine cells [15, 38, 39]. The main controversy is the distribution of other organs, especially in the brain and liver. Some researchers have found that GPR119 also exists in mouse liver, rat insular cortex gustatory insula, human brain, liver, skeletal muscle, and myocardium [33, 38, 40–42]. However, Odori S and others found that *GPR119* mRNA was not detected in esophagus, liver or cerebrum in human tissues [43]; the different results may be due to low expression levels or differences in detection conditions. More researchers should adopt more sensitive and unified detection schemes to clarify its distribution (see Fig. 1).

### GPR119 ligands

GPR119 is mainly a stimulatory G protein α-subunit (Gas)-coupled G protein-coupled receptor [44], but it seems to be related to Gai and Gaq and can interact with β-arrestin [45]. Identifying its ligands and clarifying related physiological responses are essential to treat diseases [46]. We referenced and updated the list of ligands appropriately [15, 41]. The ligands are mainly categorized into endogenous ligands (see Table 1) and synthetic agonists (see Table 2). Many studies of GPR119 agonists have focused on their aspects of promoting insulin secretion and improving glucose tolerance. However, MAFLD is correlated with the pathological factors of T2DM, and hence GPR119 agonists have potential as therapeutic agents for alleviating MAFLD.

**Table 1:** GPR119 endogenous ligands

Name	EC_50_ (μM)	Refs.
2-Oleoyl glycerol (2-OG)	2.5–17	[44] [121] [61] [62]
Oleoylethanolamide (OEA)	0.2–5	[15, 63]
N-Oleoyl-dopamine (OLDA)	3.2	[53]
Lysophosphatidylethanolamine	5.7	[36]
Lysophosphatidylinositol	5.7	[36]
Lysophosphatidylserine	> 30	[36]
Lysophosphatidic acid	> 30	[36]
Sphingosylphosphorylcholine	> 30	[36]
Oleic acid	> 1000	[36]
Palmitoyl-lysophosphatidylcholine (16:0-lysoPC)	1.6–2.1	[36]
Stearoyl-lysophosphatidylcholine (18:0-lysoPC)	3.3	[36]
Oleoyl-lysophosphatidylcholine (18:1-lysoPC)	1.5–9	[36]
5-Hydroxy-eicosapentaenoic acid (5-HEPE)	0.03–3	[52]
Palmitoylethanolamide (PEA)	0.84	[61]
Linoleoylethanolamide (LEA)	0.56–5	[61]
2-Linoleoyl glycerol	12	[61]
2-Palmitoyl glycerol	11	[61]
2-Arachidonoyl glycerol	*NA*	[61]
1-Oleoyl glycerol(1-OG)	2.8	[61]
1-Linoleoyl glycerol	36	[61]
Anandamide	*NA*	[61]
Oleamide	4.5	[53]
*N*-Arachidonoyldopamine	*NA*	[53]
*N*-Oleoyl-tyrosine	0.7	[53]
Arachidonoyl ethanolamide (AEA)	*NA*	[51]

*NA* not applicable

**Table 2:** GPR119 synthetic agonists

Name	Chemical structure	EC_50_ (μM)	Pharmacological effects in vivo and in vitro	Refs.
AR231453		0.0047–0.009	AR231453 increases the levels of cAMP, GLP-1, and insulin	[68, 71, 72]
PSN821	Structure not disclosed	*NA*	PSN821 can reduce weight and increase GLP-1 levels	[79, 122]
MBX-2982		0.0039	MBX-2982 increases GLP-1 secretion, improves blood glucose control, inhibits fat production and reduces cholesterol	[123, 124]
GSK1292263		*NA*	GSK1292263 reduces HbA1c levels and glucose excursion	[123, 125]
LEZ763	Structure not disclosed	*NA*	*NA*	[79]
JNJ-38431055		0.046	JNJ-38431055 reduces glucose excursion	[55, 76]
DS-8500a		0.0515	DS-8500a improves abnormal glucose intolerance, increases GLP-1, insulin secretion and high-density lipoprotein cholesterol concentrations, reduces total cholesterol, low-density lipoprotein cholesterol and triglyceride concentrations	[126–132]
ZYG-19	Structure not disclosed	*NA*	*NA*	[79]
AR246881		0.0097	*NA*	[76]
BMS-903452		0.014	BMS-903452 reduces glucose excursion, increases GLP-1 and insulin secretion	[79]
AR44006	Structure not disclosed	0.1704	AR44006 increases insulin secretion	[63]
AR435707	Structure not disclosed	0.0277	AR435707 increases insulin secretion	[63]
GSK-1104252A		0.05	*NA*	[79]
APD668		0.0027	APD668 reduces cholesterol, TG levels, body weight ALT and AST	[55]
ARN-II		*NA*	ARN-II enhances GLP-1 secretion, increases cAMP level	[133]
AZ1		*NA*	AZ1 enhances GLP-1 secretion, increases cAMP level	[133]
AZ2		*NA*	AZ2 enhances GLP-1 secretion, increases cAMP level	[133]
AZ3		*NA*	AZ3 enhances GLP-1 secretion, increases cAMP level	[133]
AS1269574		2.5	AS1269574 protects β cell function and alleviates disorders of glucose and lipid metabolism	[134]
AS1535907		1.5–4.8	AS1535907 protects β cell function and promotes insulin secretion	[135–137]
AS1907417		1.1	AS1907417 enhances intracellular cAMP, GSIS, and human insulin promoter activity and regulates adipogenesis	[91]
AS1669058		0.11	AS1669058 improves glucose tolerance and promotes insulin secretion	[79]
PSN119-2		0.4	NA	[76, 79]
PSN632408		1.9	PSN632408 could increase the cAMP level and insulin secretion	[138]
PSN375963		8.4	PSN375963 increases insulin and GLP-1 secretion	[40]
PSN119-1		0.5	PSN119-1 increases insulin and GLP-1 secretion	[139]
PSN119-1 M		0.2	PSN119-1 M increases insulin and GLP-1 secretion	[139]
Compound 3		1.7	Compound 3 increases insulin and GLP-1 secretion	[139]
Compound 1		0.5	Compound 1 increases insulin and GLP-1 secretion	[139]
HD0471953	Structure not disclosed	*NA*	HD0471953 can improve glucose tolerance and increase cAMP level	[83]
HD044703	structure not disclosed	0.11	HD044703 can improve glucose tolerance and enhance cAMP, GLP-1 and insulin secretion	[140]
HD0471042	Structure not disclosed	0.65–0.85	HD0471042 can improve glucose tolerance and enhance cAMP, GLP-1 and insulin secretion	[141]
ZB-16		0.00725	ZB-16 enhances GLP-1 and insulin secretion, decreases blood glucose levels and improves glucose utilization	[142]
HBK001		0.03	HBK001 promotes the release of GLP-1, improves glucose tolerance and protects islet β cell function	[90]
compound 8		0.013	Compound 8 reduces the level of blood glucose	[143]

*NA* not applicable

### Endogenous ligands of GPR119

Oleoylethanolamide (OEA), LPC, retinoic acid, palmitoylethanolamide (PEA), arachidonoylethanolamide (AEA), etc., are considered to be endogenous ligands that activate GPR119 on intestinal endocrine cells (ECCs) to activate adenylate cyclase (AC), thereby increasing the downstream cAMP and increasing the release of incretins, causing a series of physiological effects [47–51]. The rank order of the effectiveness of various ligands to activate GPR119 is first OEA, then LPC, PEA, stearoylethanolamide (SEA), and finally AEA [49]. Ryouta Kogure et al. found that the ω-3 unsaturated fatty acid metabolite 5-hydroxy-eicosapentaenoic acid (5-HEPE) also activates GPR119 with an efficacy approximately equal to that of OEA [52]. *N*-oleoyldopamine (OLDA), a lipid amide, can be extracted from the bovine striatum and has been a potent endogenous ligand for GPR119 along with other hydroxybenzyl lipid amides. The potency of OLDA is equivalent to the potency of OEA [53, 54]. In addition, some lysophospholipids and other lipid breakdown products, such as LPC, oleic acid, and 1-Oleoyl glycerol (1-OG), can activate GPR119, but because of their low potency, their activity has not been determined [55].

### LPC

For all the studied lysophospholipids, LPC produced by phospholipase A2 (PLA2) seems to be the most effective in activating GPR119 [55]. LPC activates GPR119 to cause glucose-dependent insulin release (GSIS). It has been a promising candidate for anti-T2DM [56]. The earliest discovery showing that LPC can promote insulin release was made by Metz et al. [57], who discovered various LPCs in 1986, including LPC 16:0, LPC 18:0, and LPC 18:1, all of which are present in human plasma [56]. Moreover, LPC, as a marker for a variety of liver diseases, is elevated in MAFLD, but saturated LPC is reduced in patients with advanced cirrhosis, and it is associated with mortality risk [58]. In addition, LPC can protect against hepatitis by binding to type II natural killer T cells, produce anti-inflammatory effects in inflammatory diseases, increase anti-inflammatory factor levels and reduce the production of inflammatory mediators, including interleukin-6 (IL-6) and nitric oxide (NO) [59]. In contrast, Gurunathan Murugesan et al. found that the chemotactic effect of LPC on monocyte chemotactic protein-1 (MCP-1), interleukin-8 (IL-8) and RANTES may have a pro-inflammatory effect [60]. Therefore, the role of LPC in inflammation needs further confirmation.

### 2-Oleoyl glycerol (2-OG)

Being among the most effective natural agonists of GPR119, OEA and 2-monoacylglycerols (2-MAGs), triglyceride metabolites, have been extensively studied, especially 2-OG. The study of Jeppe H. Ekberg et al. proved that in triglyceride metabolism, 2-OG activates GPR119 to promote the secretion of incretins, and when combined with GRP40 agonist, has a synergistic effect [44]. Whether 2-OG specifically activates GPR119 is not clear because of the instability of 2-OG itself. H.A. Hassing et al. first used 2-oleyl glyceryl, a 2-OG analog, in wild-type and GPR119-knockout mice and found that GPR119 improves glucose tolerance and is eliminated by GPR119 antagonists [16]. Katrine B. Hansen et al. used human GPR119-transfected COS-7 cells to confirm that 2-OG and other monoacylglycerols activated GPR119 to increase the secretion of GLP-1 and other hormones and suggested that GPR119 acts as a fat sensor [61]. Interestingly, Karen Kleberg et al. found that 2-OG formed by lipoprotein esterase (LDL) acts as a lipid signal transducer in the vascular system [62].

### Oleoylethanolamide (OEA)

OEA, as an endogenous fatty acid derivative, is a natural agonist of GPR119 [15, 63]. OEA is a peroxisome proliferator activated receptor α (PPAR-α) agonist that reduces food intake and promotes lipid oxidation [64]. In addition, OEA may reduce fat gain in high-fat diet mice by activating the GPR119 pathway [65]. Studies have shown that bile acids regulate OEA production and activate GPR119 to regulate gastric emptying and increase satiety in experimental mouse models [66]. Hilary A. Overton et al. found that GPR119 at least partially mediated the effect of OEA on food intake, and they orally administered to rats PSN632408, a new agonist of GPR19, which inhibited food intake and white fat accumulation [40]. Similarly, Flock, Grace et al. used AR6231453, a GPR119 agonist, and found that it inhibited gastric emptying through a GPR119-dependent pathway and prolonged gastric emptying time [67]. However, it is still unclear whether the gastric inhibitory effect of OEA-activated GRP119 is specific. Hong Lan et al. used GPR119-knockout mice to find that GPR119 is unnecessary for the gastric inhibitory effect of OEA [68]. Moran et al. found that the gastric inhibitory effect produced by OEA may involve pancreatic polypeptide (PPY) [50]. OEA can trigger effects similar to those observed after bariatric surgery, including reduced food intake, reduced fat mass, increased GLP-1 release, and reduced lipid levels, which are undoubtedly beneficial to patients with MAFLD [69].

### Synthetic GPR119 ligands

Because of the great attraction of targeting GPR119 to T2DM, many synthetic GPR119 agonists have appeared. Here are some of the ligands and pharmacological effects of synthetic GPR119.

### AR231453

AR231453 is the first GPR119 agonist developed by Arena Pharmaceuticals (EC50 = 0.0047–0.009 uM) [70, 71]. Chu et al. found that AR231453 strongly stimulated glucose-dependent insulin release and cAMP accumulation by testing in cells transfected with human GPR119 and rat islets, but there was almost no response in GPR119-deficient mice or those lacking GPR119 cells [68]. Also, Marty et al. found that the use of AR231453 significantly increased the release of GLP-1 from rat intestinal L cells [72]. AR231453 has been used in several pre-clinical studies on diabetes, showing that it can regulate glucose homeostasis and increase the secretion of incretins [73, 74]. It is worth noting that GPR119 expression within murine B cells may not be important for the response to hyperglycemia or the direct insulin secretion response to GRP119 agonists in isolated mouse pancreatic islets and GPR119 β-cell-deficient mice [75].

### APD597

APD597, also known as JNJ-38431055, is a synthetic GPR119 agonist. Some clinical trials are currently ongoing or completed to evaluate its pharmacokinetics, safety, tolerability, and role in obesity and T2DM. Semple et al. fount that JNJ-38431055 (3–30 mg/kg PO) significantly improved the glucose excursion of diabetic experimental rats [76]. Studies have demonstrated that oral administration of APD597 is safe and well tolerated, and it can increase the secretion of incretin and insulin and decrease incremental plasma glucose excursion during oral glucose tolerance test in T2DM patients, but the final hypoglycemic effect is not ideal [77]. In a double-blind, randomized and placebo-controlled study, oral JNJ-38431055 (2.5–800 mg) in healthy male volunteers is also safe and well tolerated, and it can increase the concentrations of GLP-1, GIP, and PYY. Compared with the placebo group, APD597 did not significantly increase insulin secretion or glucose excursion, but it had a higher insulin secretion rate in a graded glucose infusion study [78].

### AS1669058

AS1669058 (EC_50_ = 0.11 μM) is a new generation of GPR119 small molecule agonist reported by Astellas company and further improved from AS1269574 (EC_50_ = 2.5 μM) [79]. Oshima et al. found that AS1669058 dose-dependently stimulates insulin secretion in HIT-H15 cells and isolated rat pancreatic islets. Administration of 1 mg/kg of AS1669058 significantly improved the glucose tolerance of ICR mice, and administration of 3 mg/kg of AS1669058 twice a day for a week reduced the glucose level of *db/db* mice [80].

### Others

There are still many synthetic GPR119 agonists, including PSN632408, HD0471953, MBX2982, GSK1292263, and BMS903452, and some are undergoing clinical trials (see Tables 2 and 3), most of which concern their role in T2DM. It was found that PSN632408 could increase the cAMP level and insulin secretion of HEK293 cells transfected with GPR119 [81]. In 2019, Fang et al. synthesized and evaluated a series of novel fused pyrimidine derivatives as GPR119 agonists; some of these analogs (16, 19, 26, 28, 42) have high GPR119 agonistic activity [82]. Single dose administration of HD0471953 can improve the oral glucose tolerance test (OGTT) in normal C57BL/6 J mice, and increase insulin secretion and GLP-1 level. Also, HD0471953 stimulates a dose-dependent increase in cAMP levels in the HIT-T15 β cell line, and it reduces the body weight, high-density lipoprotein (HDL), LDL cholesterol, TG and epididymal fat in experimental T2DM mice [83]. Compared with normal mice, BMS903452 at the dose of 0.1–0.3 mg/kg reduced the glucose excursion by 30–40% in an OGTT, BMS903452 and a DPP-IV inhibitor synergistically regulated GLP-1 levels in a Sprague–Dawley rat model, and BMS903452 (0.03 mg/kg/day) reduced fasting blood glucose levels and increased insulin secretion in *db/db* mice [79]. BMS903452 at 0.1–120 mg was safe and tolerable to healthy subjects in a clinical trial, but no significant increase in plasma total GLP-1 level was observed in the first 24 h of treatment [84].

**Table 3:** GPR119 clinical trial agonists

Name	Condition or disease and ClinicalTrials.gov number	Sponsor	Interventions	Primary outcomes	Secondary outcomes
Oleoyl glycerol	Type 2 diabetes (NCT01043445)	Glostrup University Hospital, Copenhagen	2-Oleyl glycerol, oleic acid, vehicle	The effect of this newly discovered GPR 119 agonist on gut hormone responses, in particular GLP-1 in response to the different meals administered to the subjects	Glucose homeostasis, gall bladder contraction in response of the different meals administered to the subjects
	Type 2 diabetes (NCT02264951)	Glostrup University Hospital, Copenhagen	Tributyrin, C8-diet oil, olive oil, carrot	Plasma GLP-1 and GIP	Plasma insulin, PYY, glucose, neurotensin and cholecystokinin
MBX-2982	Type 2 diabetes (NCT01035879)	CymaBay Therapeutics, Inc	MBX-2982, sitagliptin, placebo	Absolute and percent change from baseline and placebo in mean weighted average of 14-point blood glucose levels associated with a standardized breakfast and lunch	Additional glycemic parameters
	Type 1 diabetes (NCT04432090)	Translational Research Institute for Metabolism and Diabetes, Florida	Placebo MBX-2982 No medication	Maximal glucagon concentration, total area under the curve (AUC) for glucagon and incremental AUC during hypoglycemia	
GSK1292263	Healthy volunteers (NCT00783549)	GlaxoSmithKline	An undetermined dose and ascending dose of GSK1292263	(1) Safety and tolerability parameters including adverse events, clinical laboratory, electrocardiogram, and vital signs assessments (2) Pharmacokinetic parameters, maximum observed plasma drug concentration, time to maximum observed concentration	(1) Pharmacodynamic endpoints (2) Pharmacokinetic parameters following a dose, with and without food, and bioavailability (3) Relationships between drug exposures and pharmacodynamic parameters, safety, and tolerability, as appropriate
	Healthy subjects (NCT01101568)	GlaxoSmithKline	Simvastatin, rosuvastatin, GSK1292263	(1) AUC_(0-inf)_ and C_max_ of rosuvastatin alone and in the presence of GSK1292263 (2) AUC_(0-inf)_ and C_max_ of simvastatin/simvastatin acid alone and in the presence of GSK1292263	(1) Adverse events, cardiovascular findings (blood pressure, heart rate, ECGs) and clinical laboratory values (2) PK parameters: time to maximum plasma concentration, apparent plasma terminal elimination half-life and area under the plasma concentration–time curve for rosuvastatin [AUC_(0–72 h)_] and simvastatin/simvastatin acid [AUC_(0–24 h)_] (3) PK parameter values: AUC_(0–24 h)_, Cmax, tmax and t1/2 for GSK1292263 and assessment of steady-state
	Type 2 diabetes (NCT01128621)	GlaxoSmithKline	GSK1292263, GSK1292263 matching placebo, sitagliptin	Adverse events, serious adverse events, abnormal hematology values of potential clinical importance (PCI), abnormal clinical chemistry values of PCI, etc., 39 items in total	
	Type 2 diabetes (NCT01119846)	GlaxoSmithKline	GSK1292263,GSK1292263 matching placebo, sitagliptin	Adverse events, number of participants with abnormal hematology parameters of potential clinical importance (PCI) and abnormal clinical chemistry parameters of PCI, abnormal- clinically significant electrocardiogram (ECG) findings, T_max_ and C_max_, etc. 31 items in total	Number of Participants With AEs and SAEs, T_max_,T_lag_,C_max,_ AUC_(0-t)_ and AUC_(0–24)_ etc. 13 items in total
	Dyslipidemia (NCT01218204)	GlaxoSmithKline	10/80 mg atorvastatin, GSK1292263 placebo, 100/300/800 mg GSK1292263, 10 mg ezetimibe, washout	Adverse events, serious adverse events, abnormal- clinically significant electrocardiogram (ECG) findings, etc. 41 items in total	Trough concentration, AUC_(0–24 h)_, T_max_ and C_max_ of atorvastatin metabolite (2-hydroxyatorvastatin)
PSN821	Type 2 diabetes (NCT01386099)	Prosidion Ltd	PSN821, placebo	Beta-cell function	HbA1c, fasting plasma glucose, body weight
JNJ-38431055	Healthy male volunteers (NCT00910923)	Johnson & Johnson Pharmaceutical Research & Development, L.L.C	JNJ-38431055	Safety and tolerability	Pharmacodynamic effects of JNJ-38431055 on plasma glucose and insulin, during a meal tolerance test (MTT)
	Healthy overweight or obese adult male volunteers (NCT01054118)	Johnson & Johnson Pharmaceutical Research & Development, L.L.C	JNJ-38431055, sitagliptin 100 mg, JNJ-38431055 + sitagliptin 100 mg, placebo	GLP-1 levels after a standard meal	(1) Pharmacokinetics of JNJ-38431055 administered alone and in combination with sitagliptin (2) Appetite and satiety (3) Safety and tolerability of JNJ-38431055 administered alone and in combination with sitagliptin as measured by occurrence of adverse events, ECGs, vital signs, and safety laboratory measurements ⑷ Incremental glucose changes after MTT
	Type 2 diabetes (NCT00946972)	Johnson & Johnson Pharmaceutical Research & Development, L.L.C	JNJ-38431055, placebo	Adverse events, laboratory values, vital signs, ECGs	24 h weighted mean glucose, fasting plasma glucose, glycosylated albumin, dose response, beta-cell function, incretin levels
	Type 2 diabetes (NCT00871507)	Johnson & Johnson Pharmaceutical Research & Development, L.L.C	JNJ-38431055 Dose 1/2, sitagliptin 100 mg, placebo	Incremental glucose AUC after an oral glucose tolerance test (OGTT)	Incremental glucose AUC after a MTT, beta-cell function, incretin levels, pharmacokinetics, safety and tolerability
DS-8500a	Healthy subjects (NCT03699774)	Daiichi Sankyo, Inc	DS-8500a, rosuvastatin	Maximum observed plasma drug concentration (C_max_), time of maximum observed concentration (T_max_) and area under the plasma concentration time curve (AUC) from time 0 to the last quantifiable concentration (AUC last) for single dose rosuvastatin	C_max_, T_max_, AUC from time 0 to 24 h (AUC_0-24 h_), Metabolite to parent (M:P) AUC_0-24 ratios_, Minimum observed analyte concentration that was just prior to the beginning of the dosing interval (C_trough_), C_max_ at steady state (C_max,ss_), AUC during the 24 h dosing interval (AUC_tau_), accumulation ratio (AccRatio), T_max_ at steady state (T_max,ss_)
	Healthy subjects (NCT02790684)	Daiichi Sankyo, Inc	DS-8500a	Total 14C radioactivity in urine and feces	C_max_, T_max_, AUC, number and severity of adverse events
	Healthy subjects (NCT02790671)	Daiichi Sankyo, Inc	Itraconazole, DS-8500a	C_max_, T_max_, AUC	Number and severity of adverse events, change in physical examination findings, 12-lead electrocardiogram, vital sign measurements and clinical laboratory test results
	Type 2 diabetes (NCT02685345)	Daiichi Sankyo Co., Ltd	DS-8500a 25/75 mg, placebo	Change in 24 h weighted mean glucose	Change in fasting plasma glucose, plasma glucose, glycoalbumin, serum insulin, proinsulin, C-peptide, pancreatic peptide YY3-36, GLP-1, total GIP, total glucagon, total cholesterol, HDL, LDL, TG, and derived (plasma glucose, serum insulin, C-peptide, pancreatic peptide YY3-36, GLP-1, total GIP, total glucagon) AUC, number and severity of adverse events
	Type 2 diabetes (NCT02669732)	Daiichi Sankyo Co., Ltd	DS-8500a, placebo	First-phase and second-phase secretion insulin and C-peptide	M value, M/I value, disposition index, number and severity of adverse events, plasma concentration of DS-8500a
	Type 2 diabetes (NCT02222350)	Daiichi Sankyo Co., Ltd	10/75 mg DS-8500a tablet, placebo	Change in 24-h weighted mean blood glucose	Change in 24-h weighted mean blood glucose, blood fasting plasma glucose level, blood plasma glucose level, blood insulin level, blood C-peptide level, blood active GLP-1 level, blood PYY level, blood HbA1c level, blood glycoalbumin level and postprandial plasma glucose level, number and severity of adverse events, pharmacokinetic profile of DS-8500a
	Type 2 diabetes (NCT02628392)	Daiichi Sankyo Co., Ltd	DS-8500a, placebo, sitagliptin	Change in HbA1c	Change in HbA1c, plasma glucose, AUC derived from plasma glucose, serum insulin, AUC _0–3 h_ serum insulin, AUC _0–3 h_ proinsulin, AUC _0–3 h_ C-peptide, AUC _0–3 h_ PYY, PYY, GLP-1, AUC _0–3 h_ total GIP, total GIP, AUC _0–3 h_ glucagon, glucagon, AUC _0–3 h_ 1,5 AG, 1,5 AG, total cholesterol, HDL cholesterol, LDL cholesterol and TG Proportion of subjects with HbA1c < 7.0
	Type 2 diabetes (NCT02647320)	Daiichi Sankyo, Inc	Sitagliptin 100 mg, DS-8500a 25 mg, placebo tablet, placebo capsule	Change from baseline in glycated hemoglobin (HbA1c)	Change from baseline in total cholesterol (TC), LDL-C, HDL-C, non-HDL-C, triglycerides, area under the curve 0–3 h (AUC _0–3 h_) of plasma glucose (PG), AUC _0–3 h_ of PG, Cmax, C_max_ of PG and fasting plasma glucose (FPG) Count of participants with HbA1c less than 7.0%
LEZ763	Normal healthy volunteers and patients with type 2 diabetes (NCT01619332)	Novartis Pharmaceuticals	Placebo, sitagliptin, LEZ763,	Adverse events, serious adverse events, death, pharmacokinetics of LEZ763	Area under the GLP-1 curve (AUC_0-24 h_), 2-h value of post-prandial glucose, change from baseline in fasting C-peptide, fasting insulin, fasting plasma glucose, peak glucose level following meal test, peptide YY and GIP. Peak effect (E_max_) on postprandial GLP-1
ZYG-19	CTRI/2011/12/003013 (Clinical Trials Registry—India)				
BMS-903452	Normal healthy volunteers and patients with type 2 diabetes (NCT01240980)	Bristol-Myers Squibb	BMS-903452, placebo	Safety and tolerability	Pharmacodynamic activity of the investigational drug on glucose and hormones regulating glucose metabolism, ECG parameters, percent urinary recovery (% UR), renal clearance (CLR) from plasma, C_max_, T_max_, pharmacokinetics parameter
APD668	Discontinued	Arena			
"NN"	Discontinued	Novartis			
DA-1241	Type 2 diabetes (NCT03061981)	Dong-A ST Co., Ltd	placebo, metformin, DA-1241	Safety and tolerability	C_max_, T_max,_ AUC, apparent terminal elimination half-life (t^½^), apparent total systemic clearance after oral administration (CL/F), apparent volume of distribution (V_z_/F), amount of DA-1241 excreted unchanged in the urine in each collection interval (Ae), renal clearance (CLR), cumulative percentage fraction of DA-1241 excreted unchanged in the urine (Cum Fe)
	Type 2 diabetes (NCT03646721)	Dong-A ST Co., Ltd	placebo, sitagliptin, DA-1241	12-lead ECGs, blood pressure, heart rate, body temperature, respiratory rate, physical examination, clinical laboratory testing, adverse events	C_max_, T_max,_ AUC, apparent terminal elimination half-life (t^½^), apparent total systemic clearance after oral administration (CL/F), apparent volume of distribution (V_z_/F), HbA1c, fasting insulin, glycated albumin, incremental WMG (iWMG), weighted mean glucose (WMG) etc. 21 items in total

### The relationship between the GPR119/incretin axis and MAFLD

**Fig. 2:**
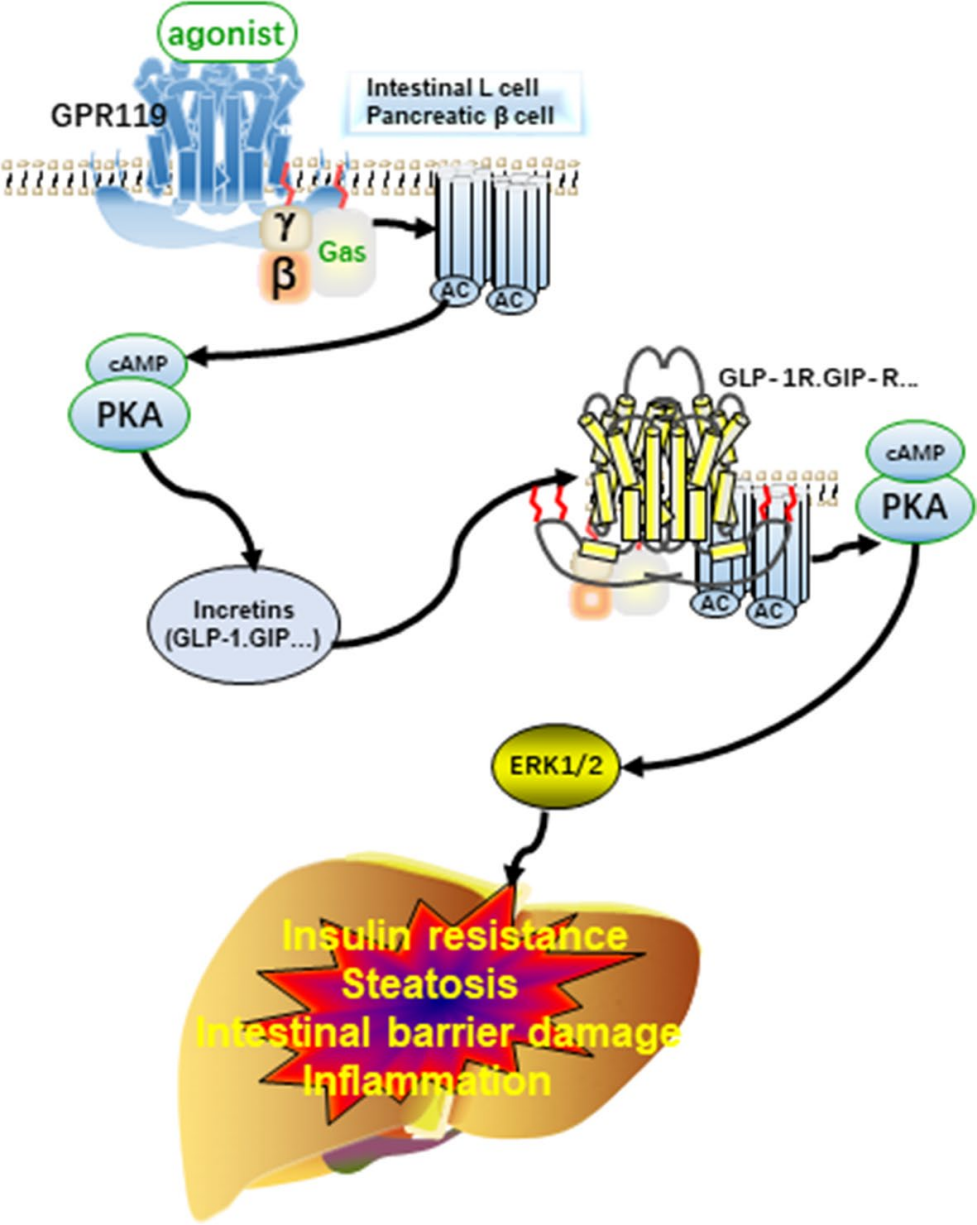
Relationship between MAFLD and the GPR119/incretin axis. When GPR119 is activated by different ligands, it leads to an increase in cAMP and combines with PKA to secrete incretins. After further activating the corresponding receptor, it can improve the disease characteristics in MAFLD through the ERK1/2 signaling pathway. AC, adenylyl cyclase. cAMP, cyclic AMP. PKA, protein kinase A. ERK1/2, extracellular signal-regulated protein kinase 1 and 2

The secretory response of incretins is due to the activation of enteroendocrine cells after food intake by the intestinal system. The principle incretins are GIP and GLP-1, produced by K cells in the proximal gut and L cells in the distal gut, respectively [85]. GPR119 activated by various factors can promote the secretion of incretins, which may attenuate MAFLD, including its effect on sugar metabolism, lipid metabolism, inflammation, and the intestinal micro-ecosystem. This process may involve the cAMP/protein kinase A (PKA)/cAMP response element-binding protein (CREB) and extracellular signal-regulated protein kinase 1 and 2 (ERK1/2) pathways. When GPR119 is activated by a variety of endogenous and exogenous factors, heterotrimeric G-protein activates adenylate cyclase (AC) and then activates PKA/mitogen-activated protein kinase kinase1/2(MEK1/2)/ERK1/2 protein sequentially to play a physiological role. The primary source of AC is adenosine triphosphate (ATP) mediated by the Class-III AC/ADCY (adenylate cyclase) family. PKA enhances intracellular calcium influx through the phosphorylation of voltage-dependent calcium channel (VDCC), thus increasing insulin secretion [86] (see Fig. 2).

### The GPR119/incretin axis enhances glucose metabolism

Insulin resistance and hyperglycemia accompany glucose metabolism disorders in MAFLD patients, and hyperglycemia increases the production of intracellular reactive oxygen species (ROS), which destroy mitochondrial function and lead to hepatocyte apoptosis, which aggravates MAFLD [87–89]. The GPR119/incretin axis may have unique advantages for attenuating hyperglycemia. Huan et al. discovered that HBK001, a new candidate GPR119 agonist and DPP4 inhibitor, promotes the release of GLP-1, improves glucose tolerance and protects islet β cell function [90]. Furthermore, Kim et al. found that HD0471953, a GPR119 agonist, attenuates disorders of insulin sensitivity and blood sugar control [83]. Yoshida et al. found that AS1269574 protects β cells function and alleviates disorders of glucose and lipid metabolism by reducing triglyceride and non-esterified free fatty acid (NEFA) levels in the body [91]. Studies have shown that AS1907417 in three cell lines, HEK293 cells, NIT-1 cells, and MIN-6-B1 cells, enhances intracellular cAMP, GSIS, and human insulin promoter activity and regulates adipogenesis [91]. Therefore, further attention should be paid to its unique advantages in lipid metabolism.

### The GPR119/incretin axis enhances lipid metabolism

MAFLD patients often present with reduced glycolysis and very low-density lipoprotein (VLDL) output due to insulin resistance and other factors; thus, TG levels in the liver increase, and TG accumulates in liver cells, causing liver cell degeneration, inflammation and insulin resistance in the liver to worsen, forming a vicious cycle. Studies have shown that lipid infusion increases the expression of GPR119 in volunteers [92]. The increase in cAMP caused by the GPR119/incretin axis can protect β cells from oxidative damage and lipid-induced apoptosis [93, 94]. The agonist-induced GPR119/incretin axis can reduce *stearoyl-coA desaturase -1(SCD-1)* mRNA levels by attenuating insulin resistance, leading to decreased liver adipogenesis [95]. Kim et al. used DA-1241, a GPR119 agonist, to inhibit adipogenesis and reduce steatosis through the inactivation of sterol regulatory element-binding protein-1c (SREBP-1c), the key factor of adipogenesis mediated by AMPK signaling [96]. Similarly, MBX-2982/GSK1292263, after the second phase of a clinical trial on synthetic ligands, was confirmed to function through the same mechanism as in liver cells and HepG2 cells to inhibit fat production and reduce cholesterol [38]. Bahirat et al. found that the use of APD668 alone in an MAFLD mouse model induced by a high-fat diet (HFD) can lower cholesterol and TG levels, reduce body weight and improve insulin resistance. In particular, when used together with linagliptin, a DPP4 inhibitor, APD668, may reduce liver steatosis, attenuate weight gain, and reduce alanine aminotransferase (ALT) and aspartate aminotransferase (AST) levels by inhibiting lipogenic-related gene (*SREBP-1c*, *FASN* and *SCD-1*) levels. The authors speculate that the direct activation of GPR119 and the prolonged time of GLP-1 may cause its effect. Using a DPP4 inhibitor or GLP-1 agonist alone can reduce fat formation, activate the AMP-activated protein kinase (AMPK) pathway and enhance insulin sensitivity. The combined use of the two agents has a synergistic effect, suggesting their combination in the treatment of MAFLD [97–100]. Nitika Arora Gupta et al. used HepG2 and Huh7 cells to prove that GLP-1 can act on glucagon-like peptide-1 receptor (GLP-1R), another GPCR, thereby reducing the content of TG in the liver [101]. Therefore, the GPR119/incretin axis may have a synergistic effect with other GPCR receptors. In addition, Psichas et al. found that chylomicrons, lipoprotein particles that transport exogenous TG, hydrolyze TG into long-chain fatty acids (LCFAs) and monomers through an independent mechanism with the participation of lipoprotein esterase (LPL), which GPR119 recognizes to enhance the release of incretins [26]. The GPR119/incretin axis not only has advantages in regulating TG content but is also quite effective in lowering cholesterol. Recently, Yan-Wei Hu et al. found that oxidized LDL induces the expression of lincRNA-DYNLRB2 to upregulate GPR119 and ABCA1, an essential protein in anti-cholesterol transport, which increases apoA-I-mediated cholesterol efflux and inhibits related inflammatory factor expression [102]. Importantly, GPR119 agonists such as APD668 and GSK1292263 were also found to reduce cholesterol levels, although the mechanism is not yet clear [79, 100].

### The GPR119/incretin axis and inflammation

In MAFLD, hepatic steatosis and intestinal microbial secretions can activate Kupffer cells (KCs) to release proinflammatory factors, such as TNF-α, IL-6, and interleukin-1β (IL-1β), which cause inflammation [103]. Magdalena Grill et al. found that an increase in the endogenous ligand OEA of GPR119 may exert an anti-inflammatory effect in inflammatory bowel disease [104]. Similarly, as mentioned above, the endogenous GPR119 ligand LPC also has anti-inflammatory effects. Notably, the effect of GPR119/incretins on inflammation seems to be achieved by indirectly enhancing GLP-1. By using the normal mouse model of stably expressed rAd-GLP-1, Y.-S. Lee et al. found that GLP-1 can reduce not only fat accumulation but also the expression of pro-inflammatory factors, such as TNF-α, IL-6, and macrophage infiltration and inflammatory pathways, thereby inhibiting inflammation [105]. GLP-1 can inhibit IL-1, interleukin-18 (IL-18), and nuclear factor-kappa B (NF-κB) to reduce inflammation in adipose tissue [106]. In summary, its correlation with inflammation deserves further experimental exploration.

### The GPR119/incretin axis regulates gut microbes

The intestine has an enormous surface area and diverse functions. As one of the important organs of the human body, the intestine is host to microbes that act as media for communication with the outside world and are indispensable for human health [107, 108]. Recently, Chepurny et al. found that AS1269574 acts as a dual agonist to activate GPR119 and TRPA1 cation channels to promote calcium influx and the release of incretin hormones, suggesting the possibility that the dual effects of the intestinal liver axis and quantum channels can be controlled [109]. Cohen et al. used bioinformatics to find that the part encoded by the *N*-acyl amide gene of human symbiotic bacteria interacts with GPR119 by mimicking human lipid signaling molecules, such as 2-OG, showing a way to treat metabolic diseases thought to regulate intestinal microbes [110]. Fitriakusumah et al. found that MAFLD is significantly associated with the overgrowth of intestinal flora [111]. The overgrowth of intestinal flora in MAFLD patients can cause changes in the permeability of the intestinal mucosa and the destruction of tight junction structures, resulting in lipopolysaccharides (LPS) and other substances entering the blood, and they interact with Toll-like receptors through NF-kB and other pathways, producing inflammatory mediators and triggering chronic inflammation and insulin resistance (IR) [112, 113]. Lund et al. found that activation of endogenous GPR119 promotes enteroendocrine cells to enhance the release of serotonin. Although it may have a pro-inflammatory effect, in most cases, the latter can protect the intestinal barrier and secrete intestinal protective mucus [114, 115]. After activation of GPR119, the release of GLP-2 has a protective effect on the intestinal barrier and inflammation. Patrice D Cani et al. found that the use of GLP-2 antagonists abrogated the improvements to the intestinal barrier induced by intestinal microbes, suggesting a specific effect between intestinal L cells and intestinal microflora [108]. Recently, Png CW et al. discovered that a gut microbe, *A. muciniphila*, is related to several diseases with increased intestinal permeability [116, 117]. Amandine Everard et al. used this bacterial treatment to improve endotoxemia, inflammation, and insulin resistance related to metabolic disorders. It has been proven that this bacterial treatment can increase 2-OG levels. Importantly, 2-OG can also activate GPR119 [118].

### Future prospects and challenges

Metabolex and Sanofi-Aventis signed a massive investment agreement to develop the latest GPR119 pharmacological agent. Although there are currently approved injections of liraglutide and exenatide that directly target GLP-1, the discovery of GPCRs has led to opportunities for innovative development of oral active drugs [119], and there are many clinical GPR119 agonists (see Table 3). GPR119 is highly expressed in the digestive system, such as the gastrointestinal pancreas, and there is little evidence that it is expressed in the human central nervous system; thus adverse side effects in the nervous system are avoided. Another problem for GPR119 treatment is the development of related candidate compounds. Although there are currently excellent specific GPR119 agonists, their efficacy is another competing element affecting the future drug development market, and there are excellent comments and discussions about this aspect of drug development [79]. Although most of the current clinical trials of GPR119 have focused on treating T2DM, and some of the experimental results are not ideal, the safety and tolerability of MBX-2982 and PSN821 are worthy of recognition, and GLP-1 secretion is increased. Therefore, as long as chronic metabolic diseases such as MAFLD continue to exist and no specific drug is found, comprehensive investigation into potential effects of GPR119 and well-designed clinical trials still need to be conducted. In addition, the GPR119 sequence of rodents and humans are different, so there may be differences in the translation of results based on various rodent experimental models to clinical practice, which is also an important factor that should be considered [120].

## Conclusion

The GPR119/incretin axis may have a protective effect on MAFLD through a series of physiological effects by attenuating insulin resistance, reducing fat production, reducing dietary intake, reducing weight gain, increasing cholesterol outflow, and interacting with intestinal microbes. However, as a Gas-coupled receptor, GPR119 has a single pathway of action that may provide only a small contribution to the attenuation of metabolic diseases, and there may be synergy between receptors of different coupling pathways. Therefore, further research is urgently needed in the future to determine the effect that may be related to the GPR119/incretin axis and convert it into an effective clinical MAFLD treatment plan.

## Data Availability

Not applicable.

## Abbreviations

MAFLD: Metabolic (dysfunction)-associated fatty liver disease
NAFLD: Non-alcoholic fatty liver disease
T2DM: Type 2 diabetes mellitus
GPCR: G protein-coupled receptor
GLP-1: Glucagon-like peptide-1
GIP: Glucose-dependent insulinotropic peptide
cAMP: Cyclic AMP
GLP-2: Glucagon-like peptide-2
PYY: Peptide YY
TG: Triglyceridemia
DPPIV: Dipeptidyl peptidase 4
LPC: Lysophosphatidylcholine
MECA: Melanocortin, endothelial differentiation gene, cannabinoid, and adenosine
Gas: G protein α-subunit
OEA: Oleoylethanolamide
PEA: Palmitoylethanolamide
AEA: Arachidonoyl ethanolamide
ECCs: Endocrine cells
AC: Adenylate cyclase
SEA: Stearoylethanolamide
5-HEPE: 5-Hydroxy-eicosapentaenoic acid
OLDA: *N*-oleoyl dopamine
1-OG: 1-Oleoyl glycerol
PLA2: Phospholipase A2
GSIS: Glucose-dependent insulin release
IL-6: Interleukin-6
NO: Nitric oxide
MCP-1: Monocyte chemotactic protein-1
IL-8: Interleukin-8
2-OG: 2-Oleoyl glycerol
2-MAG: 2-Monoacylglycerol
LDL: Lipoprotein esterase
PPAR-α: Peroxisome proliferator activated receptor α
OGTT: Oral glucose tolerance test
HDL: High-density lipoprotein
PKA: Protein kinase A
CREB: CAMP response element-binding protein
ERK1/2: Extracellular signal-regulated protein kinase 1 and 2
MEK1/2: Mitogen-activated protein kinase kinase1/2
ATP: Adenosine triphosphate
VDCC: Voltage-dependent calcium channel
ROS: Reactive oxygen species
NEFA: Non-esterified free fatty acid
VLDL: Very low-density lipoprotein
HFD: High-fat diet
LPL: Lipoprotein esterase
KCs: Kupffer cells
TNF-α: Tumor necrosis factor alpha
LPS: Lipopolysaccharides
IR: Insulin resistance
SCD-1: Stearoyl-coA desaturase -1
SREBP-1c: Sterol regulatory element-binding protein-1c
HFD: High-fat diet
ALT: Alanine aminotransferase
AST: Aspartate aminotransferase
AMPK: AMP-activated protein kinase
GLP-1R: Glucagon-like peptide-1 receptor
LCFAs: Long‐chain fatty acids
LPL: Lipoprotein esterase
KCs: Kupffer cells
IL-1β: Interleukin-1β
IL-18: Interleukin-18
NF-kB: Nuclear factor-kappa B
LPS: Lipopolysaccharides
IR: Insulin resistance
LEA: Linoleoylethanolamide
PCI: Potential clinical importance
ECG: Electrocardiogram
MTT: Meal tolerance test
OGTT: Oral glucose tolerance test
C_max_: Maximum observed plasma drug concentration
C_max,ss_: C_max_ at steady state
T_max_: Time of maximum observed concentration
T_max,ss_: T_max_ at steady state
AUC: Area under the plasma concentration time curve
AUC last: From time 0 to the last quantifiable concentration
M:P: Metabolite to parent
AUC_0-24__ h_: AUC from time 0 to 24 h
AUC_tau_: AUC during the 24 h dosing interval
AccRatio: Accumulation ratio
TC: Total cholesterol
AUC _0__–__3__ h_: Area under the curve 0–3 h
FPG: PG and fasting plasma glucose
CLR: Renal clearance
iWMG: Incremental WMG
WMG: Weighted mean glucose
Vz/F: Apparent volume of distribution
CL/F: Apparent total systemic clearance after oral administration
t½: Apparent terminal elimination half-life
